# 单细胞水平解析T细胞大颗粒淋巴细胞白血病免疫谱系特征

**DOI:** 10.3760/cma.j.cn121090-20241125-00479

**Published:** 2025-05

**Authors:** 苛 黄, 乐乐 张, 晨 邱, 若难 李, 昱灿 沈, 伟望 李, 虹 潘, 珍 高, 力维 方, 雅婧 初, 卫平 袁, 均 施

**Affiliations:** 1 中国医学科学院血液病医院（中国医学科学院血液学研究所），血液与健康全国重点实验室，国家血液系统疾病临床医学研究中心，细胞生态海河实验室，天津 300020 State Key Laboratory of Experimental Hematology, National Clinical Research Center for Blood Diseases, Haihe Laboratory of Cell Ecosystem, Institute of Hematology & Blood Diseases Hospital, Chinese Academy of Medical Sciences & Peking Union Medical College, Tianjin 300020, China; 2 天津医学健康研究院，天津 301600 Tianjin Institutes of Health Science, Tianjin 301600, China

**Keywords:** 白血病，大颗粒淋巴细胞, 免疫细胞, 差异基因, 通路富集分析, Leukemia, large granular lymphocytic, Immune cell, Differentially expressed genes, Pathway enrichment analysis

## Abstract

**目的:**

单细胞转录组层面解析T细胞大颗粒淋巴细胞白血病（T-LGLL）免疫谱系变化，探索其致病机制。

**方法:**

收集2019年6月至2020年12月中国医学科学院血液病医院收治的5例T-LGLL患者治疗前后及3名健康志愿者外周血，应用10×Genomics技术进行单细胞转录组建库测序，比较患者与健康志愿者的免疫细胞差异基因并进行通路富集。

**结果:**

通过对67 237个免疫细胞分析，发现T-LGLL患者疾病状态下：①效应CD8^+^ T细胞数量增多、细胞毒性及增殖能力增强，免疫抑制治疗有效后效应CD8^+^ T细胞增殖能力及效应功能均下降（*P*<0.05）；②调节性T（Treg）细胞比例减少且凋亡增加，免疫抑制治疗有效后Treg细胞比例升高，凋亡通路下调（*P*<0.05）；③抗原提呈细胞（APC）功能增强，单核细胞、树突状细胞均可富集到抗原合成与呈递通路，B细胞抗原结合能力增强，富集到与T细胞活化相关通路（*P*<0.05）；④自然杀伤（NK）细胞的毒性杀伤功能减弱，但对T细胞的调节能力增强（*P*<0.05）。

**结论:**

T-LGLL患者存在特征性免疫谱系，表现为免疫稳态的失衡，突出的特征为效应CD8^+^ T细胞异常活化伴数量增加，Treg细胞数量减少且功能失调；APC及NK细胞正向调控T淋巴细胞激活、分化和增殖。

## Abstract (en)

**Objective:**

To investigate alterations in the immune lineage of T-cell large granular lymphocytic leukemia（T-LGLL）at the single-cell transcriptome level and to elucidate its pathogenic mechanisms.

**Methods:**

Peripheral blood samples were collected from 5 T-LGLL patients before and after treatment（from June 2019 to December 2020）and 3 healthy controls at the Institute of Hematology & Blood Diseases Hospital, CAMS & PUMC. Single-cell transcriptome sequencing libraries were prepared and sequenced using 10× Genomics technology. Differentially expressed genes in immune cells were compared between patients and healthy donors, followed by pathway enrichment analyses.

**Results:**

Profiling 67,237 immune cells revealed that, in T-LGLL: 1）Effector CD8+ T cells exhibited increased numbers, enhanced cytotoxicity, and greater proliferative capacity. Following effective immunosuppressive therapy, both the proliferative capacity and effector functions of these cells significantly decreased（*P*<0.05）. 2）The proportion of regulatory T（Treg）cells was reduced, accompanied by increased apoptosis. After effective immunosuppressive therapy leading to remission, Treg cell proportions increased, and apoptotic pathways were downregulated（*P*<0.05）. 3）Antigen-presenting cells（APCs）showed enhanced functionality. Monocytes and dendritic cells were enriched in antigen synthesis and presentation pathways, while B cells displayed increased antigen-binding capacity and were enriched in pathways related to T-cell activation（*P*<0.05）. 4）Natural killer（NK）cells exhibited attenuated cytotoxic function but demonstrated an enhanced regulatory capacity over T cells（*P*<0.05）.

**Conclusion:**

T-LGLL patients present a characteristic immunological profile marked by an imbalance in immune homeostasis. This profile includes abnormal activation and expansion of effector CD8^+^ T cells, and a reduction in Treg cell numbers accompanied by functional impairment. Furthermore, APCs and NK cells were found to positively regulate T-lymphocyte activation, differentiation, and proliferation.

T细胞大颗粒淋巴细胞白血病（T-LGLL）是一类以细胞毒性T淋巴细胞扩增为主要特征的罕见淋巴增殖性疾病[1]。其发病机制尚未明确，目前研究主要聚焦于细胞毒性T淋巴细胞，普遍认为抗原持续激活多种信号通路，从而使终末效应T大颗粒淋巴细胞持续单克隆扩增，进而导致患者出现骨髓受累、脾大、关节痛等临床表现。约40％的患者合并自身免疫性疾病相关特征，提示该疾病可能存在免疫稳态失衡[2]–[3]。为了更全面解析T-LGLL的病因和发病机制，本研究借助单细胞测序技术，从转录组层面对比T-LGLL患者治疗前后及与正常人群之间的差异，解析T-LGLL患者的免疫细胞功能，为解释其致病机制、探索诊疗技术提供新思路。

## 材料与方法

一、样本来源

设置5例符合T-LGLL诊断标准[1]–[2]的患者为实验组，留取其治疗前后外周血；3名健康志愿者（2名男性、1名女性，年龄分别为55岁、45岁和33岁）为对照组，留取外周血；提取外周血单个核细胞（PBMC）。所有样本均来自中国医学科学院血液病医院（中国医学科学院血液学研究所）。本研究经中国医学科学院血液病医院伦理委员会批准（批件号：QTJC2025014-EC-1）。

二、制备单细胞样本

将新鲜分离及复苏的PBMC用含2％胎牛血清的PBS（Sigma公司，美国）重悬，通过流式细胞仪分选4′, 6-二脒基-2-苯基吲哚（DAPI，Biolegend公司，美国）阴性活细胞群。用适量体积的含有0.4％牛血清白蛋白（Sigma公司，美国）的D-PBS重悬细胞，等待上机。

三、构建测序文库

构建文库由北京诺禾致源科技股份有限公司协助，详细步骤参照10×Genomics官网（https://www.10xgenomics.com/support/cn）提供的说明书：Chromium Single Cell V（D）J Reagent Kits with Feature Barcoding technology for Cell Surface Protein。总结为六个步骤：①GEM（Gel Bead-In-EMulsions）捕获单细胞并裂解出mRNA；② mRNA纯化与反转录，cDNA扩增；③构建5′转录组文库；④ cDNA靶向富集可变区（多样性）连接区［V（D）J］区域与文库构建；⑤构建表面蛋白文库；⑥ Illumina平台测序。

四、数据分析

①质控与清洗：采用R软件的Seurat包对Cell Ranger分析结果中的表达矩阵按一定条件再次筛选，对原始数据进行质量控制，去除低质量片段。②细胞聚类与注释：分析各类免疫细胞的基因表达数据，通过比较上述基因与已知各类细胞标志基因、HCL数据库、ABC数据库、Cellmarker数据库、Panglao DB数据库记录的标志基因确定每个细胞群。通过识别差异基因实现细胞聚类。这一过程有助于揭示不同细胞群体之间的相关性和差异性。同时，利用标志基因对这些细胞群体进行详细的注释，以便更好地理解每个群体的生物学特性。③差异基因分析：使用Seurat的Find Markers函数进行差异分析，参数为min.pct＝0，logfc.threshold＝0；差异筛选阈值设置为|log2FC|>0.25，*P*<0.05。将测序结果中各群免疫细胞在患者疾病状态下的上调和下调基因进行可视化处理。④功能富集：将得到的上调和下调的基因通过GO/KEGG分析进一步发现各群免疫细胞在不同通路上的变化，该过程使用R包clusterProfiler（R软件）。

五、统计学处理

应用R 4.2.2软件进行数据处理和分析。计量资料采用*M*（范围）表示，单细胞转录组样本分析采用了FDR多重检验的方法，差异分析采用Mann Whitney *U*检验及*t*检验方法，*P*<0.05为差异具有统计学意义。

## 结果

一、患者基本信息

研究共纳入了5例患者，3例男性、2例女性，中位年龄45（43～68）岁。详见表1。所有患者均接受了一线免疫抑制治疗，并进行了单细胞转录组测序。例2接受环孢素（3 mg·kg^−1^·d^−1^）治疗7个月无效（无法脱离红细胞输注），改为环磷酰胺（100 mg/d）治疗4个月达血液学完全缓解。例3和例4经环磷酰胺治疗6个月后均有效。患者免疫抑制治疗后外周血样本测序包括：例2环孢素治疗无效状态，例2、3、4环磷酰胺治疗后缓解状态。

**表1:** 5例T细胞大颗粒淋巴细胞白血病患者的基本信息

例号	性别	年龄（岁）	病程（月）	HGB（g/L）	ARC（×10^9^/L）	ANC（×10^9^/L）	ALC（×10^9^/L）	PLT（×10^9^/L）	LGL计数（×10^9^/L）	STAT3突变	LGL免疫分型
1	男	50	22	71	60.00	0.83	7.25	362	1.61	有	CD3^+^CD8^+^；CD45RA^+^；CD7^+^；CD5^dim^CD7^dim^；CD16^dim^
2	男	43	2	79	56.80	0.92	1.82	173	0.98	有	CD3^+^；CD57^+^
3	男	54	5	80	52.30	0.98	3.17	161	2.10	无	CD3^+^CD8^+^；CD5^+^CD7^+^；CD45RA^+^；Perforin^+^；GraB^+^
4	女	56	36	60	10.00	1.02	4.25	348	2.58	无	CD3^+^CD8^+^；CD5^+^CD7^+^；CD45RA^+^；Perforin^+^；GraB^+^
5	女	68	12	51	10.00	0.97	2.35	150	1.51	无	CD3^+^CD8^+^；CD57^+^；CD45RA^+^；Perforin^+^GraB^+^；CD5^dim^CD7^dim^

**注** 病程：从出现症状到明确诊断且接受治疗的时间；ARC：网织红细胞绝对值；ANC：中性粒细胞绝对计数；ALC：淋巴细胞绝对值；LGL：大颗粒淋巴细胞

二、免疫细胞亚群分析

免疫细胞亚群的聚类情况如图1A所示，共分为15群细胞，并根据样本的采集时间点将其分为4组：健康对照（3例健康志愿者）、免疫抑制治疗前（未接受免疫抑制剂治疗的5例患者）、免疫抑制治疗无效（例2接受环孢素治疗无效状态）、免疫抑制治疗有效（例2、3、4接受环磷酰胺治疗后缓解状态）。针对不同免疫细胞亚群分析结果如下。

**图1:**
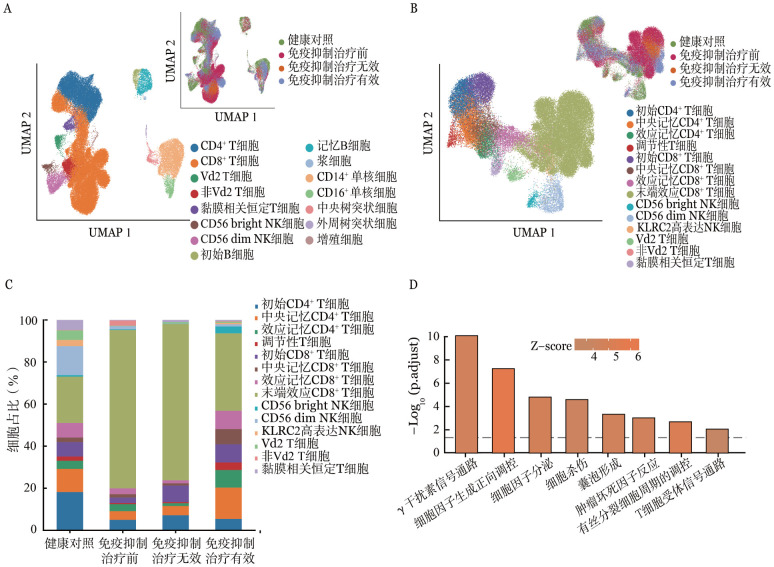
免疫细胞亚群聚类情况及CD8^+^ T细胞的转录组变化 **A** UMAP图展示各群免疫细胞聚类和注释（左下为分群，右上为分组），左下角UMAP图指所有免疫细胞的分群，右上角4种颜色分别代表了健康对照、免疫抑制治疗前、免疫抑制治疗无效和免疫抑制治疗有效；**B** UMAP图展示T细胞、NK细胞聚类及注释（左下图为分群，右上图为分组）；**C** 柱状堆积图展示4组样本中T、NK细胞的各群细胞占比；**D** 柱状图展示患者CD8_TE细胞群中上调差异基因GO/KEGG富集结果

1. CD8^+^ T淋巴细胞亚群：相比于健康对照组的CD8^+^ T淋巴细胞比例为（25.35±6.25）％，患者免疫抑制治疗前组的比例显著增多［（72.22±11.24）％，*P*＝0.001］，而免疫抑制治疗有效组比例明显减少［（39.53±14.52）％，*P*＝0.011］。免疫抑制治疗前组末端效应CD8^+^ T（CD8_TE）细胞占比高于健康对照组（75.3％对20.4％，*P*＝0.001）和免疫抑制治疗有效组（75.3％对34.0％，*P*<0.05）（图1B、C）。细胞周期分析结果表明，免疫抑制治疗前组G2/M期CD8_TE细胞的比例为（22.40±6.32）％，明显高于健康对照组［（8.82±2.93）％，*P*<0.05］和免疫抑制治疗有效组［（11.16±2.43）％，*P*<0.05］。

进一步对免疫抑制治疗前组与健康对照组中上调表达的478个差异基因进行通路富集，发现其主要富集于细胞杀伤、囊泡形成、细胞因子释放以及T细胞受体等信号通路中，表明CD8_TE细胞的毒性作用增强（图1D）。

免疫抑制治疗有效组CD8_TE细胞的比例明显降低，对比免疫抑制治疗有效组与免疫抑制治疗前组CD8_TE细胞的差异基因，发现245个基因表达减低，并且主要富集于γ干扰素（IFNγ）生成、细胞周期调控、T细胞活化、细胞杀伤调控等通路。提示免疫抑制治疗有效后CD8_TE细胞群在数量、毒性、效应功能以及调控能力等方面均发生了变化。

2. Treg细胞群：通过对健康对照组与免疫抑制治疗前组调节性T（Treg）细胞的比例和差异基因分析发现，免疫抑制治疗前组Treg细胞比例低于健康对照组［（0.56±0.27）％对（2.07±1.04）％，*P*<0.05］，基因集富集分析（GSEA）结果表明免疫抑制治疗前患者Treg细胞群凋亡通路明显上调，且氧化磷酸化过程显著下调（图2A）。而在免疫抑制治疗有效后，Treg的比例恢复［（3.64±1.29）％对（0.56±0.27）％，*P*＝0.002］。GSEA显示相比免疫抑制治疗前，免疫抑制治疗有效后Treg细胞群的氧化磷酸化通路上调，而凋亡通路和炎症反应通路均呈现出下降趋势（*P*<0.05，图2B）。

**图2:**
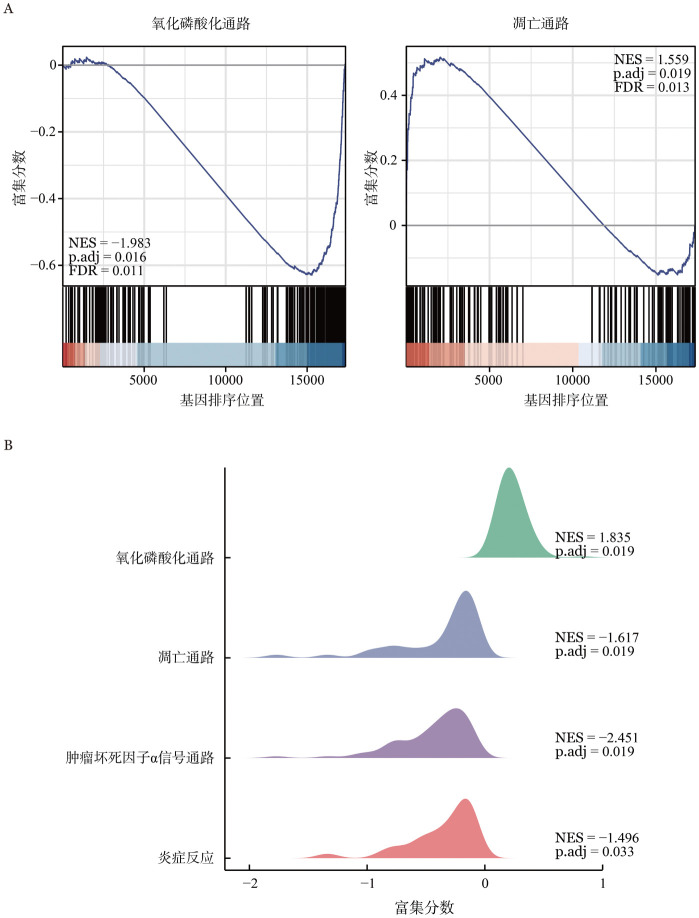
调节性T（Treg）细胞的转录组变化 **A** 健康对照组与免疫抑制治疗前组Treg细胞群基因集的GSEA富集结果；**B** GSEA山峦图展示免疫抑制治疗有效后Treg细胞群基因富集结果

3. 抗原呈递细胞（APC）：通过对患者免疫抑制治疗前和健康对照的单核细胞及树突状细胞的差异基因分析，选取了中央、外周树突状细胞，CD14^+^、CD16^+^单核细胞的差异基因，对上调基因取交集，得到185个在至少2种细胞群中上调的基因（图3A）。对这些基因进行富集分析，可富集到抗原提呈及固有免疫相关通路上调。

**图3:**
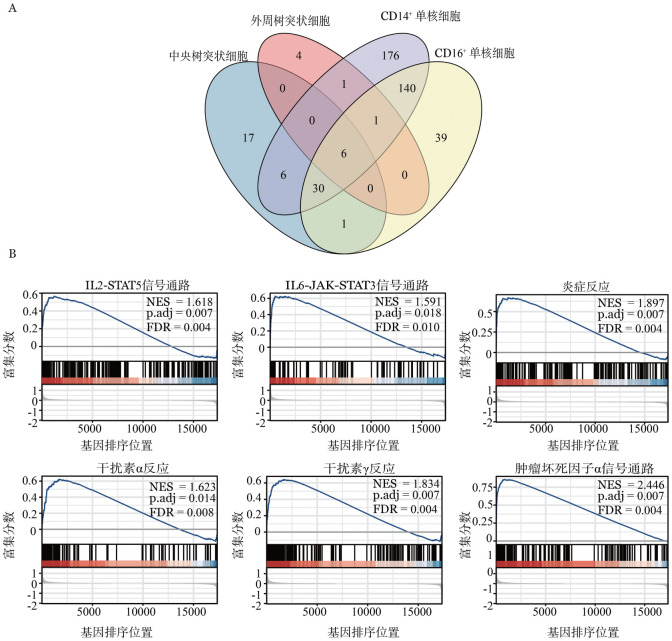
患者免疫抑制治疗前较健康志愿者的抗原提呈细胞转录组变化 **A** CD14单核细胞、CD16单核细胞、pDC、cDC四群细胞在患者中上调的差异基因交集韦恩图；**B** 两组B细胞群差异基因集GSEA富集分析结果

通过对免疫抑制治疗前组和健康对照组的B细胞转录组分析，我们发现185个差异基因中，81个基因表达上调。进一步GSEA分析发现，患者B淋巴细胞群IFNγ、肿瘤坏死因子（TNF）通路上调、炎症反应增强、STAT相关通路上调（*P*<0.05，图3B）。

4. 自然杀伤（NK）细胞：通过对免疫抑制治疗前组与健康对照组NK细胞的差异比较，发现耗竭相关基因（LAG3、TIGIT）表达升高，而且与抗原呈递相关基因（HLA-DR/DP/DQ）表达也升高，但与杀伤功能相关基因（GZMA、PRF1、GZMB、GNLY）表达下降（*P*<0.05，图4A）。进一步GO/KEGG富集分析显示，免疫抑制治疗前的NK细胞中与细胞毒性有关的通路下调但与T细胞调控相关的通路及抗原合成等通路上调（*P*<0.05，图4B）。

**图4:**
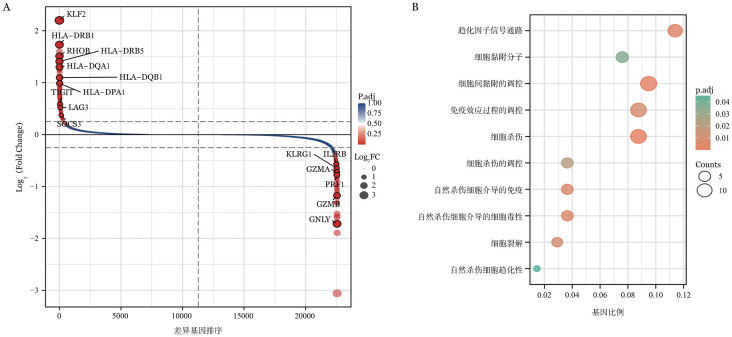
自然杀伤（NK）细胞的转录组变化 **A** 差异排序图显示免疫抑制治疗前与健康对照NK细胞群差异基因结果（上调304个基因，下调150个基因）；**B** 气泡图显示患者免疫抑制治疗前NK细胞群下调差异基因GO/KEGG富集结果

## 讨论

既往研究表明T-LGLL显著特点是克隆性T大颗粒淋巴细胞克隆性增殖[4]，本研究的单细胞转录组数据也证实了疾病状态下效应CD8^+^ T细胞数量增多，这一变化也导致了免疫细胞群的比例失衡，打破免疫平衡状态。进一步的分析表明，这些效应CD8^+^ T细胞不仅在数量上占据了优势，而且在执行其免疫功能时也表现得更为活跃。既往单细胞转录组研究[5]–[6]结果表明T-LGLL患者相比于健康供者，其CD8^+^ T细胞数量增多，其中效应CD8^+^ T细胞表现出较强的扩增能力，并且细胞毒性与耗竭相关基因呈现上调，与本研究结果相符。研究提示效应CD8^+^ T细胞数量异常增加和功能增强可能是导致T-LGLL发病的重要因素之一。

Treg细胞占CD4^+^ T细胞总数的5％～10％，能够调控T细胞的增殖和活化[7]，维持免疫系统平衡，其参与多种免疫性疾病的发生发展，如再生障碍性贫血和GVHD[8]–[9]。T-LGLL疾病状态下，Treg细胞数量的减少和功能的异常可能导致免疫系统无法有效地抑制过度的免疫反应，从而导致免疫失衡。本研究在T-LGLL的治疗过程中观察到免疫抑制治疗有效组Treg细胞的比例恢复，氧化磷酸化通路上调、凋亡通路下调，表明Treg细胞代谢功能增强与功能恢复。以上提示Treg细胞在T-LGLL病理过程中起着关键作用，恢复Treg细胞的数量和功能可能有助于改善患者的病情，为治疗提供新思路。

T细胞识别抗原具有主要组织相容性复合体（MHC）限制性，主要跟APC密切相关，包括B淋巴细胞、单核/巨噬细胞和树突状细胞[10]。以往研究表明APC可以通过多种机制介导慢性GVHD的发生[11]。本研究T-LGLL患者的CD14阳性及CD16阳性单核细胞内与抗原识别和呈递相关的基因表达水平显著上调，这些细胞通过增强抗原加工提呈能力，可能调控T细胞激活及分化进程。此外，固有免疫相关信号通路也表现出上调的趋势，提示在T-LGLL疾病状态下，患者固有免疫系统可能被激活，增强机体对病原体的早期反应能力。基因差异分析结果表明B细胞在T-LGLL疾病中可能也发挥了更为积极的作用，尤其是在对抗原的识别和呈递过程中。综上，T-LGLL患者体内APC的抗原呈递功能和对T细胞活化分化调控功能得到了显著增强。这种增强可能是导致T细胞异常活化和扩增的潜在机制之一，从而在疾病发展过程中发挥了重要作用。

NK细胞是固有免疫系统中的关键细胞类型，主要职责是抵御病原体的侵袭以及消灭发生癌变的细胞。NK细胞并不依赖于特异性抗原识别受体来执行其功能，而是具备直接杀伤能力，能够迅速识别并消灭目标细胞。NK细胞可分为CD56 bright和CD56 dim。这两种亚型在功能上各有特点，其中CD56 bright亚型的细胞表现出更强的细胞毒性，而CD56 dim亚型的细胞则在分泌IFNγ方面更为活跃。以往研究表明，NK细胞不仅参与固有免疫反应，还能够通过多种机制对T细胞的免疫反应产生影响[12]–[14]。本研究发现在T-LGLL疾病状态下，NK细胞中与细胞毒性相关的基因表达水平有所降低。然而，尽管其杀伤功能相对减弱，但在调节T细胞活化、分化和增殖方面的能力却有所增强，抗原呈递功能也有所提升。上述研究数据提示，在T-LGLL患者中，NK细胞群体可能通过调控免疫微环境稳态发挥更强的免疫调节功能。

综上所述，本研究发现T-LGLL患者免疫系统存在稳态失衡，不仅局限于CD8^+^ T细胞的改变，还包括了APC、NK细胞、Treg细胞等免疫细胞数量和功能变化。多种免疫细胞群体的功能紊乱可能是推进T-LGLL疾病发生与进展的关键因素。但本研究样本量相对有限，机制验证方面还比较欠缺，后续可能需要扩大队列结合功能实验进一步解析疾病的机制并推动精准治疗策略的开发。
